# Influential Factors in Remote Monitoring of Heart Failure Patients: A Review of the Literature and Direction for Future Research

**DOI:** 10.3390/s21113575

**Published:** 2021-05-21

**Authors:** Sashini Senarath, Geoff Fernie, Atena Roshan Fekr

**Affiliations:** 1 The Kite Research Institute, Toronto Rehabilitation Institute—University Health Network, University of Toronto, Toronto, ON M5G 2A2, Canada; geoff.fernie@uhn.ca (G.F.); atena.roshanfekr@uhn.ca (A.R.F.); 2 Institute of Biomedical Engineering, University of Toronto, Toronto, ON M5S 3G9, Canada

**Keywords:** remote monitoring, telemedicine, heart failure, data fusion

## Abstract

With new advances in technology, remote monitoring of heart failure (HF) patients has become increasingly prevalent and has the potential to greatly enhance the outcome of care. Many studies have focused on implementing systems for the management of HF by analyzing physiological signals for the early detection of HF decompensation. This paper reviews recent literature exploring significant physiological variables, compares their reliability in predicting HF-related events, and examines the findings according to the monitored variables used such as body weight, bio-impedance, blood pressure, heart rate, and respiration rate. The reviewed studies identified correlations between the monitored variables and the number of alarms, HF-related events, and/or readmission rates. It was observed that the most promising results came from studies that used a combination of multiple parameters, compared to using an individual variable. The main challenges discussed include inaccurate data collection leading to contradictory outcomes from different studies, compliance with daily monitoring, and consideration of additional factors such as physical activity and diet. The findings demonstrate the need for a shared remote monitoring platform which can lead to a significant reduction of false alarms and help in collecting reliable data from the patients for clinical use especially for the prevention of cardiac events.

## 1. Introduction

The function of the heart is to pump blood to the lungs and all the body’s tissues through a specific sequence of contractions of its four chambers. Heart failure (HF) is a condition where the heart muscle is weakened, so that the pumping action is not strong enough to supply the cells with enough blood, especially during increased activity. This can result in fatigue, shortness of breath, and coughing in some cases. To compensate, the heart will enlarge and will pump faster to increase the heart’s output [1]. The body will also compensate by narrowing the blood vessels to maintain a high enough blood pressure and diverting blood away from less important organs such as the kidneys. HF is more common in individuals who are overweight, have had a heart attack, and people who are aged 65 or older [2]. Treatment options for HF depend on the severity and type and can range from quitting smoking and losing weight to surgery. HF is now seen as a risk factor for other conditions such as stroke, high blood pressure, and atrial fibrillation [3]. The 2019 Report on Heart, Stroke, and Vascular Cognitive Impairment also reported that patients with HF are 2.6 times more likely to experience cognitive impairment [4].

Heart failure remains the leading cause of death worldwide, accounting for 31% of reported annual mortality [5]. According to the Report on Health of Canadians by the Heart and Stroke Foundation, about 600,000 Canadians are living with HF, resulting in direct costs of more than $2.8 billion per year in Canada [6]. In addition, 50,000 new cases are diagnosed with HF in Canada each year. According to Heart Failure statistics in Canada, more than 800 people per 100,000 aged 40 years and older lived with HF in the Yukon Territory in 2016, which was the highest rate in 2016 in Canada, whereas Prince Edward Island (PEI) had the lowest rate of 468 per 100,000 [7]. On average 570 people per 100,000 aged 40 years and older lived with HF in Canada in 2016. Although the Yukon Territory had the maximum HF incidence rate, this province reported the minimum mortality rate due to HF in the same year. On average 6,136 patients per 100,000 aged 40 years and older died due to HF in Canada in 2016 [7].

## 2. Review Methodology

**Figure 1:**
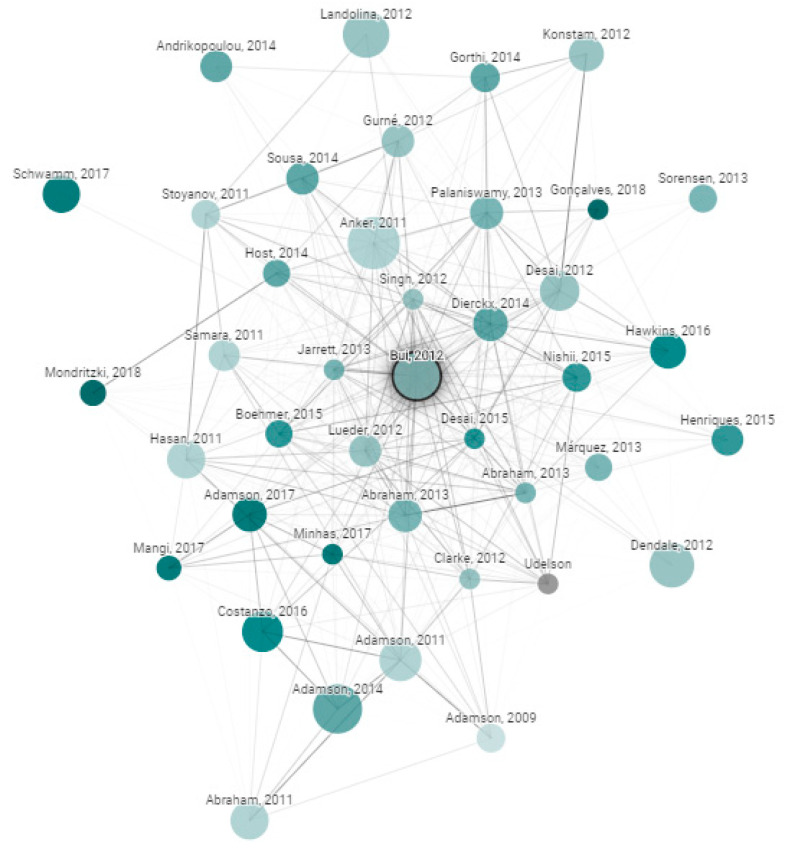
Connected papers graph sample.

Databases that were searched included IEEE, PubMed, and MDPI. Search engines that were used included Google Scholar, Microsoft Academic, UHN’s OneSearch and the University of Toronto Robarts Library. The PICO (Population, Intervention, Comparison, and Outcomes) framework was applied. In this case, the Population refers to Heart Failure patients including both acute and chronic patients, Intervention refers to telemonitoring or remote monitoring, Comparison refers to usual care, and Outcomes include cardiac events, readmission rate, and mortality. In addition, an online tool was utilized which creates a graph with papers arranged according to their similarity [8]. This is a useful tool for researchers as similar papers are clustered together and connected with stronger lines, giving a visual demonstration of the field. For each graph, about 50,000 papers were analyzed to choose the few dozen with the strongest connections to the original paper. Original papers are the references that we want to include in our bibliography. For example, our first original paper was from Bui et al. ‘Home Monitoring for Heart Failure Management’ [9], where its associated graph is shown in Figure 1. The color, size, and relative distance are all factors that contribute to the significance of the paper as described below.

Size: the number of citations.
Color: publishing year with darker nodes representing more recent papers.
Edges: the thickness of the edges represents the level of similarity.

**Figure 2:**
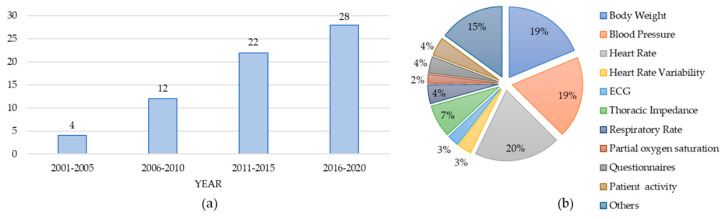
(**a**) The number of papers reviewed from 2001–2020, (**b**) the list of vital signs monitored in the reviewed papers and their percentages.

In total, we have reviewed 66 papers from 2002–2020 with the following distribution as shown in Figure 2a.

Figure 2b lists the type of vital signs monitored by the reviewed papers. Heart Rate (HR) was among the most prevalent vital sign captured in 20% of the reviewed papers following blood pressure (BP), body weight (BW), thoracic impedance (TI), and respiration rate (RR) monitored in 19%, 19%, 7%, and 4% of the papers, respectively. These results are not mutually exclusive. In other words, papers might report the use of more than one vital sign. The next section will provide an in-depth analysis of this literature.

## 3. Analysis of Literature

Individual devices tend to produce an overwhelming number of false alarms and caregivers do not have the time to sort through the noise. For example, in a pilot telemonitoring program where patients were divided into HF and acute myocardial infarction groups, clinical alerts were generated and compared [10]. A total of 1094 alerts were generated, where only 1% (10 alarms) were confirmed as clinically significant. A randomized clinical trial (RCT) with 1653 HF patients in the United States showed no significant outcome from telemonitoring for readmission or death rate after discharge [11]. Other RCTs also showed no significant benefit while comparing remote telemedical management (RTM) with usual care (UC) in all-cause mortality [12,13,14,15]. Recent results from an RCT with implanted cardiac devices showed that using weekly alerts with remote monitoring in patients with persistent atrial fibrillation increased the risk of cardiovascular hospitalization and all-cause mortality compared to the UC group [16]. The authors in [17] presented a telemonitoring system consisted of a blood pressure machine and weight scale that transmitted data to their smartphone. The main goal of this study was to test the effectiveness of digital health monitoring in real home environments. It showed that in general, patients used the weight scale less frequently than the BP machines and patients that were not readmitted to the hospital used the weight and BP monitors more often than readmitted patients. Moreover, patients older than 70 years used the monitors less often compared to younger patients. In another study with 710 patients, the authors investigated the use of an RTM on the endpoint of all-cause mortality in ambulatory patients with chronic heart failure (CHF) [12]. Their results showed that, compared to UC, the RTM method did not result in a reduction in all-cause mortality. Moreover, the published Tele-HF study led to similar conclusions, showing that neither rehospitalization rates nor mortality were affected by telemonitoring for CHF patients [11]. Although in [18] it was shown that the health status of patients significantly improved when comparing the telemonitoring group to the UC group, the difference in rehospitalization at 30 days was not significant. In another study with 1437 participants, information regarding heart rate, blood pressure, weight, and other symptoms was transmitted daily using telemonitoring equipment [19]. The combination of telemonitoring and health coaching phone calls did not reduce 180-day readmissions. Moreover, 180-day mortality and 30-day hospitalizations were not reduced. There was also low adherence to the intervention. Generally, poor adherence to remote monitoring is a main barrier in large studies [20].

**Figure 3:**
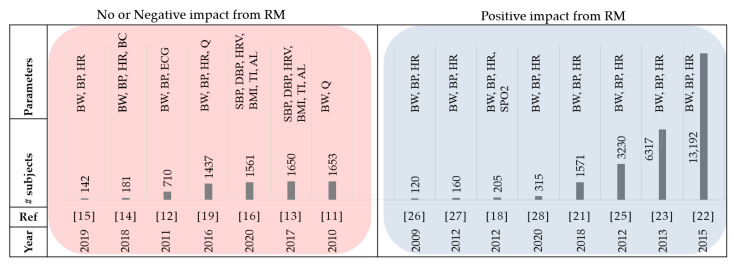
Summary of literature compared the RM vs. usual care. BW: body weight, BP: blood pressure, HR: heart rate, BC: body composition, SBP: systolic blood pressure, DBP: diastolic blood pressure, BMI: body mass index, TI: thoracic impedance, and AL: activity level.

There exist studies showing that with the implementation of a well-designed remote monitoring system, days lost due to all-cause mortality and unexpected cardiovascular hospitalizations could be reduced [21]. Recent advances in sensor technology, telecommunication and data analysis have enabled the development of precise and convenient Internet of Things (IoT) platforms for remote patient monitoring. Previous systematic reviews suggested that non-invasive telemonitoring can result in the significant reduction of HF hospitalizations and all-cause mortality compared to the UC group and was shown to be beneficial in terms of both participant satisfaction and cost [22,23,24]. In addition, initial findings from the UK Whole Systems Demonstrator trial, considering 3000 patients, indicated that using telehealth had profound clinical benefits for various conditions such as HF [25]. Among the patients hospitalized for worsening heart failure during the study period in [26], the telemonitoring group had a significantly shorter length of stay (median = 6.5 days) compared to control group patients (median = 10 days). There was a significant decrease in HF-related hospitalizations and mortality rates in the telemonitoring group where the heart rate, body weight, and blood pressure were measured [27]. The authors also showed that total hospitalization costs for the UC group were nearly twice the costs of the telemonitoring group. Moreover, the 6-month study of a phone-based telemonitoring program called Medly which facilitates patient self-care and clinical decisions, demonstrated a decrease in the number of HF-related hospitalizations and all-cause hospitalizations by 50% and 24%, respectively [28]. Figure 3 summarizes the papers reviewed in this section with increasing number of subjects.

Different vital signs are monitored by remote monitoring platforms to predict HF events. In this review paper, we categorize the current studies according to the type of monitored vital signs, as outlined in Table 1. In each sub-section, we have reviewed papers that discussed the role of the specific physiological data for predicting HF events and reported the impact of the individual parameter if it was available. Each section will include a visual presentation to emphasize the significance of each variable, independently. It is worth mentioning that different studies may use similar vital signs but may use different algorithms for detecting HF events. This review paper aims to provide the readers with comprehensive information on the results of the various studies using remote monitoring for HF patients. In addition, we will list the challenges and limitations associated with current studies and trials in the next sections.

### 3.1. Weight Monitoring

Thirty-five reviewed papers used body weight (BW) measurement as one important factor for HF patients. Sudden increases in body weight can be a sign of fluid accumulation and can be examined to detect deterioration [29]. The Canadian Cardiovascular Society (CCS) Guidelines for the Management of Heart Failure, the Heart Failure Association of America (HFSA), and the European Society of Cardiology (ESC) guidelines all recommended daily monitoring of BW for HF patients. According to CCS Guidelines, unstable patients’ weights should be closely monitored, and patients should go for a medical visit if there is a rapid gain of greater than 1.5–2 kilograms (kg) in 24 h. Likewise, weight loss without cause is one of the symptoms of advanced HF (e.g., cardiac cachexia). Therefore, the weight should be monitored daily in the morning for patients with HF that have fluid retention or congestion not easily controlled with diuretics [30]. The ESC recommends that patients that gain more than 2 kg of weight in 3 days should notify healthcare professionals and increase their diuretic dose [31]. Based on recommendations by HFSA, HF patients need to control their water and sodium intake after an increase of more than 0.9 kg (2 lbs) in their weight in 1 day, or more than 1.8 kg (4 lbs) over a week. The results in [32] indicated that BW monitoring is a simple, but important method to check for fluid retention, as opposed to non-key measures such as urine volume and leg edema. The results from the longitudinal study after 1-year body weight monitoring (WM) of 66 patients showed that WM-belief is highly associated with WM compliance [32]. This demonstrates that people who think they can manage their disease by WM will take their weight measurements more regularly [32]. A study by Sherter et al. showed that simply providing knowledge to patients about the important role of daily BW measurements could improve the adherence to weight measurements from 67% to 93% [33]. Therefore, it is important to reiterate to patients the significance of daily weight recordings so that they are encouraged to regularly do it of their own volition. According to Kozier and Erb, significant changes in weight over a short period of time (a difference of 2 kg in less than a week), may suggest acute fluid changes [34]. Each kilogram of weight gained is equivalent to 1 L of fluid gained. Quick changes of total BW from 5% to 8% can indicate moderate to severe fluid volume deficits or excesses. To obtain accurate daily weight measurements, certain criteria must be met. These include:

(1) The patient should be weighed at the same time each day (before breakfast and after the first void),
(2) The patient should be wearing the same or similar clothing, and
(3) The patient should be weighed using the same scale.

**Figure 4:**
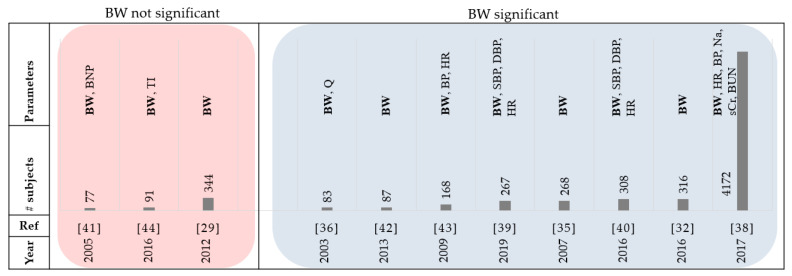
Summary of reviewed papers in Section 3.1. BW: body weight, BNP: B-type natriuretic peptide, HR: heart rate, SBP: systolic blood pressure, DBP: diastolic blood pressure, TI: thoracic impedance, and BUN: blood urea nitrogen.

Chaudhry et al. [35] found that all 134 patients hospitalized for HF, experienced gradual weight gain beginning about 30 days before the event. There was a statistically significant difference in daily weight changes between the control and study groups (*p* < 0.001). In addition, based on a clinical study conducted in the hospital, weight gain was reported as a predictor of 30-day rehospitalization or death [36]. In a study by the Acute Heart Failure Committee of the Heart Failure Association (HFA) of the European Society of Cardiology (ESC), daily weight monitoring was listed as one of the priorities of research for acute HF patients [37]. Though the relationship was non-linear, the ASCEND trial demonstrated an overall increase in risk for patients who gained weight and a decrease in risk for patients who lost weight [38]. After risk adjustment, increasing BW was associated with a 16% increase (per kg) in the risk of 180-day mortality, among patients who gained greater than 1kg post-discharge. In studies by Joshi et al. [39] and Koulaouzidis et al. [40], it was also confirmed that the weight is the most predictive feature in the univariate analysis for HF hospitalization. On the other hand, the results from different threshold-based methods for weight and B-Type Natriuretic Peptide (BNP) indicated poor sensitivity and inconsistent specificity for clinical deterioration in HF patients [41]. The use of BW in predicting HF events is directly affected by the type of algorithm. For example, the HeartPhone algorithm that used 7-day moving averages on the daily BW data was compared to guideline weight thresholds [42]. In predicting HF events, there was a significant increase in sensitivity in the HeartPhone algorithm (82%) compared to guideline thresholds of 2 kg over 2–3 days (21%) and a ‘rule of thumb’ threshold of 1.36 kg over 1 day (46%). Similar to the results by Chaudhry et al. [35], the findings in Zhang et al. [43] demonstrated that generally there was an increase in weight before HF hospitalizations; however, significant weight gain was only found in 20% of patients in the period before hospitalization. The authors in [44] stated that it is difficult to determine whether an increase or decrease in BW is a definite sign of deteriorating condition or if it is simply due to small fluctuations in weight as it is common for healthy adults to have daily variations of between 0.5–1.5% of their BW [45]. Therefore, not only does the real time measurement of daily BW need to be considered, but post-processing techniques will also need to be explored further. Figure 4 and Table 2 summarize the review papers in this section.

### 3.2. Thoracic Bio-impedance Monitoring

**Figure 5:**
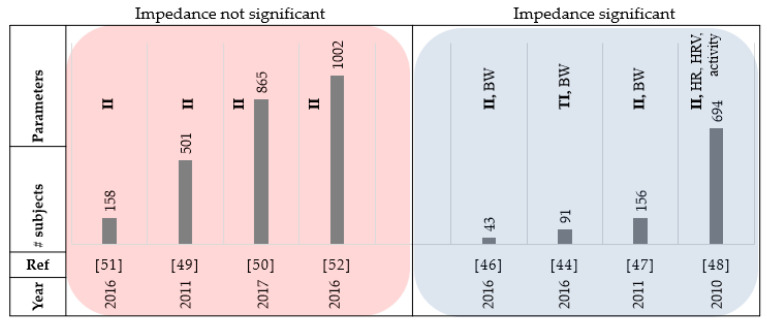
Summary of reviewed papers in Section 3.2. BW: body weight, HRV: heart rate variability, HR: heart rate, and II: intrathoracic impedance.

Fourteen of the papers reviewed used thoracic bio-impedance as a parameter. A study that investigated the relationship between daily BW and Intrathoracic Impedance (II) in HF patients reported that the false-detection rate could be decreased to clinically sufficient levels using the combination of both parameters [46]. Patients in this study were told to weigh themselves in the morning after using the washroom and before breakfast. In the case of an individual parameter, intrathoracic impedance showed higher diagnostic performance in the prediction of HF-related events compared to BW. The authors in [44] evaluated algorithms using daily BW measurements and transthoracic bio-impedance (TI) to predict HF decompensation. The weights of the patients were logged in the morning before eating breakfast, using a weight scale (Philips Medical Systems), with an accuracy of ±0.1 kg. They concluded that the data from bio impedance could predict events better than weight data considering 90 patients over 10 months. Gudmundsson et al. [46] also compared the changes in intrathoracic impedance and BW for 45 patients, 30 days before any major (HF hospitalization) or minor event. The sensitivity of intrathoracic impedance for HF-related events was reported as 83.3% (95% CI: 71.7 to 91.0) and for BW, 43.9% (95% CI: 31.9 to 56.7) considering the ESC rule. Gyllensten et al. compared different algorithms and guideline-based rules using BW in predicting HF hospitalizations [44]. Considering the ESC guideline, monitoring daily BW provided high specificity (87%) and low sensitivity (13%) which might be due the inability to account for gradual weight increases, compared to moving average algorithms that can decrease variability in BW measurements. Using the HFSA guideline of BW gain of 2 lbs or more in a day resulted in a sensitivity of 67% and specificity of 56%. This paper also concluded that transthoracic impedance provides more accurate indication of upcoming decompensations when compared to BW measurements. In a randomized trial, intrathoracic bio-impedance monitoring performed better than daily BW measurement [47]. The authors in [48] combined different parameters including night heart rate, patient activity, heart rate variability, fluid index, AF duration and ventricular rate, percentage of ventricular pacing each day, and ICD shocks to predict clinical deterioration of HF in patients with systolic left ventricular dysfunction. 72% of evaluations had more than 2 (out of 8) parameters triggered and the remaining 28% were caused by fluid index ≥100. This highlights the important role of monitoring the fluid level derived from thoracic impedance and the fusion of different parameters to increase the rate of prediction. Albeit useful, some studies [49,50,51,52] showed that the thoracic impedance provided low sensitivity in early detecting HF and did not reduce risk of HF hospitalization. The substantial number of false-positive alerts in such a system could cause a burden to informal and formal caregivers. Figure 5 and Table 3 summarize the reviewed papers in this section.

### 3.3. Blood Pressure Monitoring

**Figure 6:**
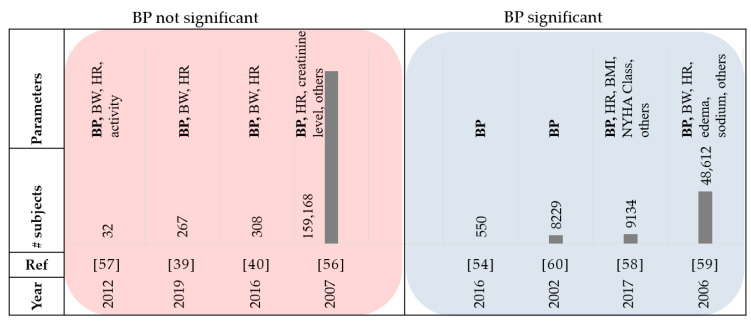
Summary of reviewed papers in Section 3.3. BW: body weight, HR: heart rate, BP: blood pressure, and BMI: Body Mass Index.

Thirty-five papers that were reviewed used blood pressure monitoring as a factor. High blood pressure is one of the common factors associated with cardiovascular disease [53]. In the CHAMPION trial, patients were randomly assigned to either the control group or the treatment group where daily pulmonary artery pressures (using an implanted device) were monitored [54]. The results showed that the readmission rate for HF was reduced in the treatment group by 33% when compared to the control group. Though this demonstrated that monitoring pulmonary artery pressures has significant long-term benefits in lowering HF hospitalization rates, other studies have shown that the systolic and diastolic blood pressure measurements alone are not an accurate indicator of HF [40,55,56,57]. HF may cause either hypertension or hypotension. In a study by Susan it was reported that about 25% of patients with HF events had systolic blood pressure (SBP) greater than 160 mmHg, while less than 10% were hypotensive [55]. This aligns with the results from [57] which reported that blood pressure did not offer any additional value in predicting HF events. The study in [56] evaluated the sequential patterns in clinical characteristics for HF hospitalization during 2002–2004. They concluded that there was no substantial change of blood pressure level in HF patients over time [56]. The studies in [39] and [40] also concluded that the SBP alone is not accurate enough for the prediction of HF events. However, when combined with weight data, they provide accurate prediction models. Considering invariant analysis proposed by [39], it was also observed that the performance of the features based on BP in HF prediction was lower than performances of BW and features derived from HR. An observational study with 9134 patients and 1-year follow-up data demonstrated that low SBP was a predictor for mortality in HF patients with a baseline left ventricular ejection fraction (LVEF) of less than 50% [58]. Based on the study conducted by Gheorghiade et al. among 48,612 patients with HF, 50% had SBP higher than 140 mmHg at the time of admittance into the hospital [59]. The SBP was greater than 140 mmHg in 38% of patients with left ventricular systolic dysfunction (LVSD). Greater SBP was correlated to substantially less in-hospital mortality. In other words, the incidence of death was observed only on 1.7% of patients with SBP greater than 161 mmHg [59]. It was shown that the lifetime risk for congestive HF patients with BP higher than 160/100 mmHg is twice the patients with BP less than 140/90 mmHg, highlighting the significance between hypertension and long-term risk of congestive HF [60]. Figure 6 and Table 4 summarize the review papers in this section.

### 3.4. Heart Rate Monitoring

**Figure 7:**
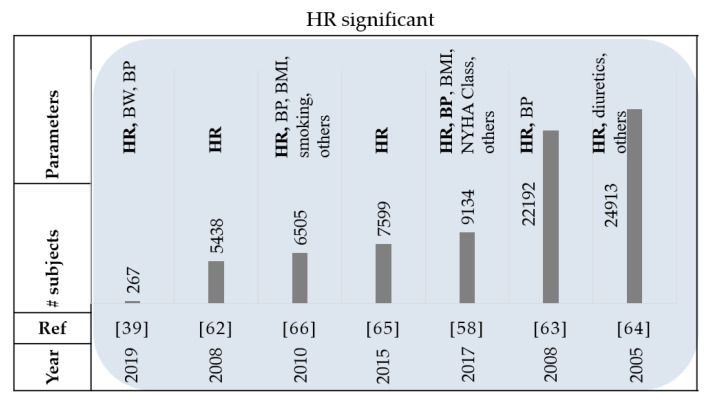
Summary of reviewed papers in Section 3.4. BW: body weight, HR: heart rate, BP: blood pressure, and BMI: Body Mass Index.

Thirty-seven papers reviewed used heart rate as a parameter in their studies. Monitoring heart rate (HR) is another part of standard clinical practice for patients with HF [61]. A study on the BEAUTIFUL trial showed that higher HR was associated with a greater occurrence of HF hospitalization and cardiovascular death [62]. Patients with a baseline resting HR of 70 beats per minute (bpm) or greater had a 53% increase in adjusted relative risk for hospitalization. Other studies have also supported this finding by concluding that an increase in resting HR can be associated with higher risk in patients with HF [58,63,64,65]. In the SHIFT study by Böhm et al., it was also confirmed that high HR is a risk factor in HF patients [66]. In the CHARM program, a time-updated HR was analyzed which refers to the most recent HR value obtained from a clinic visit to calculate the temporal short-term changes of heart rate occurring in between visits [65]. Analyzing the data categorically, they found that for a decrease of 10bpm or more, there was an associated 17% lower risk of hospitalization for HF or cardiovascular death and 15% lower risk of all-cause mortality in comparison to the ‘no change in HR’ group. In other words, their findings demonstrated that changes in HR over time can predict outcomes in patients with chronic HF. In a recent study, authors investigated whether including HR and BP information to weight data would be useful in the prediction of HF decompensation [39]. Considering 267 subjects, they found that BW was the most predictive feature in the univariate analysis. However, additional information can be obtained from the medium to short-term changes (≤8 days) in BP and for classifying the risk of decompensation in patients with HF. This means that for a window of eight days the HR-based features outperform the best weight-based feature. In a pilot program by Pereira et al., 21 HF patients were enrolled to monitor their BW, BP, HR, and heart rhythm [10]. The BW and HR were shown to have the most frequent alarm triggers in the confirmed clinical alerts. Figure 7 and Table 5 summarize the review papers in this section.

### 3.5. Respiration Monitoring

**Figure 8:**
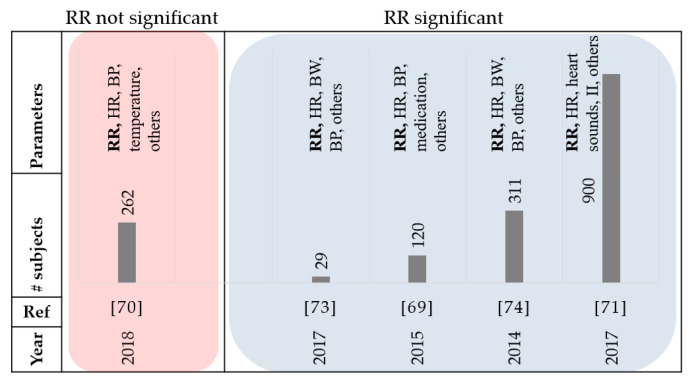
Summary of reviewed papers in Section 3.5. RR: respiration rate, BW: body weight, HR: heart rate, BP: blood pressure, and BMI: Body Mass Index.

Eight papers that were reviewed used respiration rate. Respiratory distress is a common sign in patients with HF [67,68]. A study by Goetze et al. reported that the daily minimum, maximum, and median Respiration Rate (RR), were significantly increased during the 30-day period before an HF event compared to the baseline [69]. Therefore, daily monitoring of respiration rates may be a valuable addition for the management of HF patients. In [70], the authors reported that RR increased by 18% in patients that were admitted to the intensive care unit (ICU) compared to the total population of the study. In addition, a 33% increase in RR was reported in patients who required mechanical respiratory support due to severe AHF, and 44% increase in patients who eventually died, however RR alone was not able to identify patients regarding their HF severity. In another study, at least a week before admission, over half the patients reported symptoms of worsening shortness of breath, although few indicated acute worsening during the days leading up to a hospitalization [36]. These studies suggest that respiration may be a significant factor in early prediction of HF decompensation and earlier intervention may prevent hospitalization. The MultiSENSE study also included RR data to propose an algorithm for predicting the HF events [71,72]. The RR data could help in detecting the rapid shallow breathing trends that were related to shortness of breath. In 2016, a study was conducted to monitor patients with HF at home with the implementation of a contactless under-the-mattress monitoring system [73]. The random forest classification was used to organize the information by readmission status. They concluded that readmitted patients were associated with a higher average heart rate and respiration rate, with greater respiration variability. The average RR monitored overnight was the best indicator of readmission. A study by Shoaib et al. suggests that patients with less serious symptoms seem to have a worse prognosis [74]. Out of the 42% of patients categorized as ‘short of breath at rest’ (SOBAR) in their study, 31% died; while out of the 56% classified as ‘comfortable at rest but breathless on slight exertion’ (CARBOSE), 47% died. They also reported that only 2% of HF patients were considered as ‘not short of breath’ at admission, and therefore the relevance of respiratory rate as a physiological variable in HF patients remains significant. Figure 8 and Table 6 summarize the review papers in this section.

## 4. Discussions and Future Work

Home-Health technology provides cost-effective solutions, convenient in-place monitoring, and increases the consistency of care delivery for HF patients. However, the dream of technology providing home healthcare has not become a reality for several reasons. Consumers cannot afford or are unwilling to spend as much on health technology as hospitals so the devices must be very inexpensive. There are no clinical engineering departments in people’s homes so installation and maintenance requirements must be minimal.

**Figure 9:**
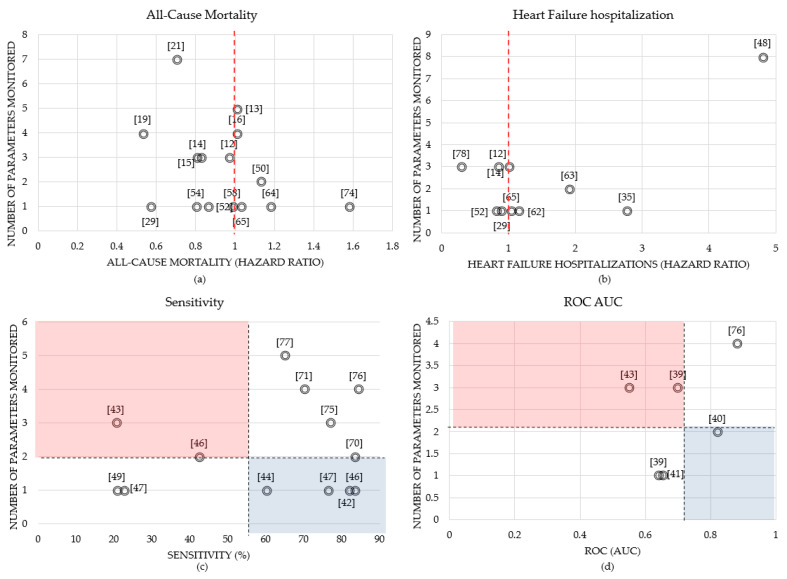
Comparison of (**a**) all-cause mortality, (**b**) heart failure hospitalizations, (**c**) sensitivity, and (**d**) ROC AUC (receiver operating characteristic area under curve) to the number of parameters monitored in various studies.

Although different studies used similar vital signs with different alarm management algorithms for detecting the HF events, it is observed that the combination of the parameters will provide more accurate detection rates compared to an individual parameter, regardless of the type of the algorithm. For example, in a study that aimed to optimize the alarm management of a HF home monitoring system, the highest specificity achieved for an alarm was based on three or more exceeded thresholds over two consecutive days [75]. In a recent study, researchers examined the performance of an individualized analytical system for predicting rehospitalization after HF admission using a combination of physiological data such as HR, heart rate variability, accelerometer, RR, and temperature [76]. The system could identify precursors of hospitalization to HF decompensation with 85% specificity and 84% sensitivity. In [53], the researchers implemented a predictive scheme combining blood pressure, body weight, respiration rate, and heart rate. Their results suggest that the physiological data obtained can be used in the early detection of HF decompensation. The MultiSENSE study also combined several variables including respiration rate, heart rate, tidal volume, thoracic impedance, and activity to propose a threshold-based alert algorithm (HeartLogic) [71,72]. This method detected the gradual worsening of HF with a 70% sensitivity and an alert window of 34 days prior to the events. The multisensory HF monitoring algorithm developed and validated by [77] also met the pre-specified performance endpoint by combining respiration rate, activity, heart rate, posture, and bio impedance data. Figure 9 compares the number of variables monitored in each study to the calculated all-cause mortality, HF hospitalizations, sensitivity, and ROC AUC, where the data labels represent the reference number of the study.

The hazard ratio is defined as the ratio of the chance of an event occurring in the treatment group versus the chance of an event occurring in the control group. A hazard ratio of exactly 1.0 means the treatment group and control group both have the same chance of an event occurring. This is represented by the red dash line in Figure 9a,b. For example, in [19], the intervention group used remote monitoring equipment to collect blood pressure, weight, heart rate, and some symptom questions, where the adjusted hazard ratio for 30-day all-cause mortality was minimal (0.53) compared to other works shown in Figure 9a. However, the authors stated this result is more likely caused by the differences of in-hospital death after randomization, rather than the intervention. Daily weight was electronically transmitted in the intervention group in [29] where the all-cause mortality and HF hospitalization hazard ratios were 0.57 and 0.9, respectively. In addition, in [78], there was a significant improvement in the intervention group compared to the usual group, where patients used telemonitoring devices to monitor BP, weight and HR over 12 months, achieving a hazard ratio for HF hospitalization of 0.29, which can be seen in Figure 9b. This figure also shows that in [48], patients with a positive combined HF device diagnostic have a 4.8-fold increase of HF hospitalization after adjusting for clinical variables.

In Figure 9c,d, the black dash lines represent the average number of parameters monitored (which was 2) and the average sensitivity (55%) and ROC values (0.7) over all reviewed papers, respectively. The blue area emphasizes the studies that performed relatively well in predicting HF decompensation and had a low number of parameters monitored, while the red area is used to emphasize the low performance studies that used a high number of monitored variables. Using one parameter of intrathoracic impedance in [46] resulted in a sensitivity of 83.3% when predicting HF decompensation, compared to using two parameters, intrathoracic impedance, and weight, which resulted in a sensitivity of 42.4% shown in Figure 9c. The authors concluded that since pulmonary fluid accumulation with clinical decompensation may not always lead to an overall increase in weight, this may explain why there is a superior sensitivity with impedance alone. Moreover, the findings in [40] showed that the HF hospitalization prediction results that used a combination of weight and diastolic blood pressure signals achieved an AUC of 0.82 with 8-day telemonitoring data.

These studies highlight the value of fusing different vital sings for predicting HF symptoms. However, there are different challenges and limitations for measuring each physiological variable. For example, one limitation is that weight measurements are affected by clothing variation and urine retention. Although all studies mentioned that the weight measurements were performed in the morning after the first void with similar clothing, they did not use any technique to control these parameters, which may have a significant impact on the results. In addition, some weight scales recommended that the weight measurement should be done barefoot to have reliable readings, especially for measuring the body fat and hydration. Incorporating methods to confirm if the measurements are performed appropriately may reduce ambiguities in the data. Some studies, such as [39], mentioned there were days of missing data where the patient did not record a weight measurement. The authors in [39] used means of linear interpolation with the two closest data points to estimate and fill the gaps of the missed data. For short-term periods, the participants might adhere to the specific rules of data collection; however, for long-term periods (months and years), there is no guarantee that the data is collected properly. As an example, in the TEN-HMS study, compliance to the telemonitoring system was stated as a limitation [43]. The result showed that prior to the worsening heart failure deaths in patients, most of the study population became inconsistent in their measurement compliance, indicating that the issue of non-compliance may be a significant predictor of increasing HF-related death. The frequency of monitoring (daily vs. less than daily) was found to be one of the strongest factors of benefit of remote monitoring. Patient adherence to self-measuring at home was also explored in the Medly study [79]. Over a 1-year period, there was a 1.4% drop in adherence after each month, in which low adherence was stated to be due to contextual factors such as technical issues, situations that conflicted with an existing habit, and a belief that the benefits were minimal due to the inability of the system to consider the entire context of the patient’s health status. Therefore, implementing mechanisms that both monitor and encourage daily measurement along with staff enthusiasm toward using the system can help improve adherence. In another study, the nurse-led collaborative management group showed promising results with reductions in readmission rates and improved quality of life compared to usual care [78]. They suggest the effectiveness of this telemonitoring study was due to the collaborative support by nurses and healthcare workers rather than simply concentrating on physiological parameters. To incentivize hospitals to improve care quality, the Hospital Readmission Reduction Program was established, which created financial penalties for hospitals in the United States with higher readmissions [80]. Although there was a reduction of 30-day and 1-year readmissions, there was also an increase in 30-day and 1-year mortality, demonstrating that the degree of involvement of the health care workers into the remote monitoring system must be carefully considered. Incorporating incentives or penalties in a system may encourage inappropriate care strategies [80]. For example, an addition of a reward system that is dependent on the number of nurse and patient visits may increase the overall number of interactions, but the quality of care of each visit may decrease. Another challenge is that the frequency and degree of HF events can be significantly affected by missed medication or medication not taken at the right time. One study demonstrated that the most common self-identified component contributing to HF decompensation was ‘missing or skipping medication’ [36], which indicates that the subject’s adherence to prescribed medication should also be monitored. The type and doses of the medications affect the weight and possibly other vital signs.

Moreover, none of the reviewed studies controlled or recorded the water intake and diet of the patients during the study. These parameters can directly affect weight measurements. In addition, air temperature and the levels of daily activity might affect vital signs. For example, in the Rotterdam Study, the authors stated that one of their limitations was the inability to account for the degree of physical activity of each patient, resulting in possible skewing of data trends [81]. More active patients will have higher respiration rates and heart rates that may cause several false positives. There is no evidence that the data is collected in a stationary position or while walking or doing daily activities in most studies. The motion artifact should be removed from the signals which requires understanding the subject’s position and type of activity during data collection. Therefore, in addition to monitoring the vital signs for predicting the HF symptoms, a system is needed to make sure the data collection protocols are performed properly. This smart system should be able to provide information about the activity levels, water intake and nutrition, and environmental temperatures. Moreover, this system should confirm if the measurements are taken at the right time with the right conditions. Heart rate is influenced by both intrinsic and extrinsic factors such as circadian rhythm and metabolic rate and smoking, diet, stress levels and physical activity, respectively [82]. It has been shown that the circadian clock influences factors such as blood pressure and heart rate [83]. The Rotterdam study also highlights the inability to account for the circadian variation in heart rate measurements [81]. Conflicting results in several studies may be due to these limitations with the inability to consider various aspects that might affect data collection. In [84], the authors demonstrated that conflicting results in studies can also be due to differences in the content of the data that is monitored, delay and quality of transmission, and workflow to connect with the patient. They suggest future studies implement strategies to ensure a long-term transmission compliance, use a wide array of medical data for alarms and to determine whether patient contact is necessary, and allow the patient to be seen within less than one week after an event.

Another challenge is that the current studies are specific to the device models and may not necessarily be extrapolated to other devices with different combinations of parameters. The rate of diagnosis can vary between models and manufacturers. Therefore, there is a need to have a platform which can provide a hub with a unifying technology that combines multiple communication protocols to connect different types of sensors, actuators, and devices through a single interface. Table 7 summarizes the challenges discussed in this section that are associated with remote monitoring of HF patients.

## 5. Conclusions

Similar to the positive impact of remote monitoring on diabetic patients, there is a great potential for remote monitoring approaches in heart failure to improve the outcome of care. This paper presented a review of the key physiological variables that are used in remote monitoring systems of HF patients and their reliability in predicting HF decompensation. These variables included daily body weight, bio-impedance, blood pressure, heart rate, and respiration rate. Studies used these parameters with various alarm algorithms for detecting HF events and decompensation with varying degrees of accuracy and sometimes conflicting results. Regardless of the algorithm, it was shown that the combination of the parameters will provide a more accurate detection rate compared to an individual parameter. Most studies also mentioned limitations that they faced with their data collection methods that may have skewed their results and challenges with poor compliance to the outlined protocol for monitoring. To overcome these challenges and increase the reliability within studies, this paper highlights the need for more control and strict guidelines when collecting data from HF patients in their home environment. Another common challenge was the external factors that could not be taken into consideration during the study. These included the physical activity of the patient, their diet, medication habits, and device variability. For future research, when creating a remote monitoring system, it is important to incorporate several physiological variables together to get the best accuracy in the prediction of HF decompensation and events. It is also important to consider methods to both lessen the variability in the data collection and improve adherence such as daily reminders. Moreover, incorporating smart devices to manage external factors can reduce the number of unknowns in the study. Using the fusion of data of noninvasive and inexpensive devices may be a promising approach for the future direction of remote monitoring systems for HF patients. A platform that has been successfully implemented could help to reduce heart attacks for many patients who could take timely medical treatment before a cardiac event occurs. The new models created could also lead to the significant reduction of false alarm rates and therefore lessen the burden on both family members and healthcare professionals.

## Figures and Tables

**Table 1:** Summary of Parameters Monitored in Section 3.1, Section 3.2, Section 3.3, Section 3.4 and Section 3.5.

Section	Monitored Parameter	Summary
Section 3.1	Body weight	Weight was significant in most studies reviewed
Section 3.2	Thoracic bio-impedance	Thoracic bio impedance showed varying significance in the reviewed studies
Section 3.3	Blood pressure	Blood pressure showed varying significance in studies
Section 3.4	Heart rate	Heart rate showed promising results in the studies reviewed
Section 3.5	Respiration	Most studies showed that respiration monitoring was significant

**Table 2:** Summary of Reviewed Papers from Section 3.1.

Ref.	Purpose	Results
[29]	To examine whether the measurement of daily BW will lead to a reduction in cardiac hospitalizations.	Daily measurement of BW did not lead to a reduction in death or hospitalization rates.
[32]	To examine factors that may have an impact on BW monitoring compliance of congestive heart failure patients.	Highlighted the significance of a high WM-belief score with better adherence to BW monitoring compliance at baseline and after 1 year.
[35]	To determine trends in weight changes before HF hospitalization and determine if increasing weight is a risk factor for hospitalization.	At least 1 week prior to HF hospitalization, there is an associated increase in BW for patients with HF.
[36]	To identify patient responses, time course, and key factors of patients with decompensated HF.	There is an observed time window between symptom exacerbation and admission. Hospitalizations may be prevented with earlier access and intervention.
[38]	To investigate the relationships between BW changes post-discharge and in-hospital and examine the effects in patients hospitalized with acute HF.	Over 30% of patients admitted for acute HF experienced small weight gain or loss during hospitalization. Among these patients, an increase in BW was associated with greater risk of adverse outcomes.
[39]	To determine the effects of adding BP and HR to weight data on the detection of HF deterioration.	The most predictive feature class was BW in the in the univariate analysis. Adding HR and BP to weight measurements improved the classification accuracy.
[40]	To implement an algorithm that uses non-invasive TM to collect daily physiological data including BW, HR, and BP to identify patients at risk of HF hospitalization.	The combination of BW and BP in the algorithm resulted in the best predictive performance for HF hospitalization using telemonitoring data over 8 days.
[41]	To examine the performance of changes in BW and B-Type Natriuretic Peptide (BNP) in determining early decompensation in HF patients.	Both absolute and relative changes in BW and BNP were not able to successfully predict clinical deterioration.
[42]	To explore trends of daily BW in patients with high-risk HF patterns of daily BW in a high-risk HF population and to compare guidelines and individualized weight monitoring algorithms.	Different algorithms were compared in terms of predicting HF events. The HeartPhone algorithm provided the best results.
[43]	To evaluate and compare BW algorithms that use telemonitoring data to predict worsening heart failure.	When using BW to predict worsening HF, the moving average algorithm had a higher performance compared to the rule-of-thumb algorithms which had a higher number of false positives.
[44]	To examine algorithms that use BW and non-invasive transthoracic bio-impedance to predict decompensation in HF patients.	Data from bio impedance vest predicted events better than weight data.

**Table 3:** Summary of Reviewed Papers from Section 3.2.

Ref.	Purpose	Results
[44]	To examine algorithms that use BW and non-invasive transthoracic bio-impedance to predict decompensation in HF patients.	Data from a bio impedance vest predicted events better than weight data.
[46]	To investigate the relationship between daily BW and II in patients with HF	Combining BW and impedance data can decrease false-detection rate to clinically acceptable levels and achieved a better performance than BW alone.
[47]	To examine the sensitivity and false alarm rate of predicting HF events between changes in daily BW and II in HF patients.	Monitoring of II achieved a significantly higher sensitivity and lower false alarm rate in predicting of HF events compared with BW monitoring.
[48]	To evaluate the use of a combination of HF device diagnostics data in predicting clinical deterioration in HF patients with systolic left ventricular dysfunction.	Patients that had positive HF device diagnostics as specified were 4.8 times more likely to have an HF hospitalization in the next month with pulmonary signs or symptoms.
[49]	To evaluate the positive predictive value (PPV) and sensitivity of II monitoring for chronic HF patients.	Fluid index derived from II had low PPV and sensitivity at the early stages after implantation of a device.
[50]	To investigate the safety and clinical effects that remote monitoring will have on HF patients that are implanted with a CRT-D with advanced diagnostics.	For HF patients that were implanted with a CRT-D, mortality, and risk of cardiovascular or device-related hospitalizations was not reduced by remote monitoring.
[51]	To determine whether the implementation of a schedule screening and patient-centered educational approach to II measurements would result in the decrease of chronic HF hospitalizations.	There was a low incidence of chronic HF hospitalizations, especially in patients with decreased intrathoracic impedance.
[52]	To determine whether early automated alarms of fluid status using telemonitoring can reduce HF-related events in patients.	There was no significant improvement using the telemonitoring with II measurements. The automated alerts did not result in a decrease in hospitalizations.

**Table 4:** Summary of Reviewed Papers from Section 3.3.

Ref.	Purpose	Results
[54]	To examine the efficacy of the previous findings showing reductions in admissions for HF after a 6 month period of pulmonary artery pressure guided management compared with UC, over a longer time period	In HF patients with consistent symptoms after hospitalization, pulmonary artery pressure-guided HF management was shown to be more beneficial than clinical assessment alone.
[56]	To describe and evaluate patterns in characteristics, treatments, and outcomes for HF hospitalizations.	Eight variables were identified as the most important risk factors for in-hospital mortality. No substantial change in blood pressure level on HF patients over time.
[57]	To investigate the relationship between various self-measured parameters of BW and BP with data automatically transmitted by ICDs.	Patient activity, BW, and the difference between resting heart rate and mean heart rate and RHR are mutually correlated. BP did not add any value.
[58]	To examine HF-related events in ambulatory HF patients with LVEF and determine predictors for mortality.	Predictors for mortality in reduced and mid-range EF were high HR and low SBP. A lower BMI was independently related to mortality in reduced and preserved EF patients.
[59]	To examine the association between SBP at admission, clinical profile, and events in patients hospitalized for acute heart failure.	Half of the patients at admission had SBP higher than 140 mmHg. Lower SBP at admission was associated with higher in-hospital and post discharge mortality rates. Patients with higher SBP at admission had lower in-hospital mortality rates.
[60]	To determine the lifetime risk of chronic HF and examine factors that may modify remaining lifetime risk at different ages	There was a lifetime risk for chronic HF of 20% in men and women and from the lowest to highest BP, there was a 2-fold gradient in remaining lifetime risk for CHF.

**Table 5:** Summary of Reviewed Papers from Section 3.4.

Ref.	Purpose	Results
[58]	To examine HF-related events in ambulatory HF patients with LVEF and determine predictors for mortality.	Predictors for mortality in reduced and mid-range EF were high HR and low SBP. A lower BMI was independently related to mortality in reduced and preserved EF patients.
[62]	To determine whether an increase of resting HR at baseline is a predictor for upcoming cardiovascular death.	Increased HR of 70 bpm or greater is associated with an increased risk of cardiovascular outcomes in patients with coronary artery disease and LVSD.
[63]	To examine the correlation between resting heart rate (RHR) and outcomes in coronary artery disease (CAD) patients that are treated for hypertension with various RHR-lowering strategies.	Regardless of the treatment strategy, CAD patients with high baseline RHR, along with patients with very low and high follow-up RHR had an increased risk of adverse outcomes.
[64]	To examine the predictive value of HR in patients with stable coronary artery disease.	Independent of other recognized factors such as high BP, smoking and diabetes, resting HR is a predictor of cardiovascular and overall mortality.
[65]	To explore the connection between changes in HR from the previous visit, time-updated HR (the most recent HR value obtained from a clinic visit), and outcomes in chronic HF patients.	Both change in HR overtime and time-updated HR during follow-up can predict outcomes in chronic HF patients.
[66]	To determine whether HR is a risk factor for cardiovascular events in HF	HR reduction with ivabradine reduces clinical events in HF, confirming that HR is clearly a risk factor. Reduction in HR, when isolated from other cardiovascular effects, minimizes risk in direct proportion to the extent that HR is lowered.

**Table 6:** Summary of Reviewed Papers from Section 3.5.

Ref.	Purpose	Results
[69]	To investigate differences in RR leading up to HF admission and its near-term risk stratification power	A greater day-to-day variability in median respiratory rate in patients with HF was associated with an increased risk of an HF event occurring.
[70]	To investigate the predictive value of RR in identifying patients with acute HF hospitalized through the emergency department (ED)	RR and HR cannot independently identify patients to the corresponding acute HF severity level. Higher RR values were associated with intubation incidence, in-hospital death and admission to Intensive Care Unit.
[71]	To implement and evaluate a device-based diagnostic algorithm to predict HF-related events.	The sensitivity of the algorithm to HF events was 70% and there was a median early warning of 34 days prior to the event.
[73]	To examine normal and modified physiological patterns in the home, and to investigate whether the modified patterns are associated with readmissions to the hospital.	RR was the most important factor associated with HF readmission. Changes in physiological date were identified that may be associated with risk of hospital readmission.
[74]	To determine whether most patients that are hospitalized with HF are short of breath at rest.	47% of patients who were classified as CARBOSE and 31% who were classified as SOBAR died. Patients who are SOBAR were associated with an increased HR and BP at admission.

**Table 7:** Summary of Challenges Discussed for Current HF Remote Monitoring Systems.

Inaccurate weight measurements	− Clothing variation − Urine retention − Variation in time of measurement
Low adherence	− Lack of motivation − Technical issues − Forgetfulness − Conflicts with an existing habit
Effects of medication	− Type and number of doses − Missed medication − Medication not taken at the right time
Effects of nutrition intake	− Water Intake − Diet
Effects of activity	− Variation in activity levels − Posture and movement during vital signs measurements
Inaccurate heart rate	− Circadian rhythm − Smoking − Diet
Device and system issues	− Variations in device models − Delay and quality of transmission − Workflow to connect to patient − Installation and maintenance

## Data Availability

Not applicable.
